# Agenda-setting for Canadian caregivers: using media analysis of the maternity leave benefit to inform the compassionate care benefit

**DOI:** 10.1186/1472-6874-14-60

**Published:** 2014-04-24

**Authors:** Sarah Dykeman, Allison M Williams

**Affiliations:** 1 Trent University, Peterborough, ON, Canada; 2 School of Geography and Earth Sciences, McMaster University, 1280 Main Street West, L8S 4K1 Hamilton, ON, Canada

**Keywords:** Social policy, Agenda-setting, Women’s labour, End-of-life care, Maternity leave benefit, Compassionate care benefit

## Abstract

The Compassionate Care Benefit was implemented in Canada in 2004 to support employed informal caregivers, the majority of which we know are women given the gendered nature of caregiving. In order to examine how this policy might evolve over time, we examine the evolution of a similar employment insurance program, Canada’s Maternity Leave Benefit. National media articles were reviewed (*n* = 2,698) and, based on explicit criteria, were analyzed using content analysis. Through the application of Kingdon’s policy agenda-setting framework, the results define key recommendations for the Compassionate Care Benefit, as informed by the developmental trajectory of the Maternity Leave Benefit. Recommendations for revising the Compassionate Care Benefit are made.

## Introduction

Research about Canada’s Maternity Leave Benefit (MLB) has been undertaken throughout the course of its evolution, with the purpose of informing better policies for women managing the dual roles and responsibilities of paid employment and parenthood [1]. Recently, a comparable program, entitled the Compassionate Care Benefit (CCB), has been developed in Canada for the purpose of supporting employees to provide care for family members and friends at end-of-life. Given the gendered nature of caregiving, most caregivers are women [2]. For example, among all CCB claims made in 2010/11, 73.9% were made by women [3]. Informal labour in the form of family caregiving is gendered; greater than 2/3 of all of Canada’s informal caregivers are women, typically those between the ages of 45 and 60 [4]. The explanation for this gender imbalance is largely due to persistent gendered expectations that women assume domestic duties, which includes caring for ill family members, compounded with gendered beliefs that women are inherently better at and are more suited to caregiving than men. The majority of caregivers who are employed are women, and their participation in the workforce provides both social and financial support to their unpaid caregiving role, although the latter is linked with reduced mental and physical health [5]. Further, managing work responsibilities often conflicts with providing care and vice-versa; this may force caregivers who are employed to sacrifice one of their two roles and/or face the negative health outcomes of role strain, which has clear implications on occupational health and quality of life. Consequently, the need for paid leaves, such as the CCB, is essential to the health and well-being of caregivers who are simultaneously employed.

The focus of this paper is to examine the development and evolution of the MLB in Canada, using Kingdon’s agenda-setting framework, in order to understand how policy windows open to allow for the implementation of change [6]. Policy windows are opportunities for policies to be placed on the government’s agenda [6]. The research presented herein has not been conducted before, and generates a deeper understanding about the contextual landscape specific to the evolution of the MLB and how this understanding can inform the potential development of the CCB. The research employs media analysis in the examination of policy windows specific to the development and expansion of the MLB, providing a case for the development and expansion of the more recently unveiled CCB.

First, a brief literature review is presented whereby the impetus for the development of the MLB and the CCB are discussed, as well as their program features. Secondly, Kingdon’s theoretical framework is introduced, followed by an overview of the research design and methods [6]. The findings of the MLB analysis, which includes the evolutionary timeline of the policy, as well as a discussion of the pertinent policy windows, precedes a similar analysis with respect to the CCB. The discussion then compares the opening of policy windows for both programs and identifies future opportunities for policy change and program improvement specific to the CCB, as based on the trajectory of the comparatively longstanding MLB.

### Background: understanding the impetus for MLB and CCB policies

Since World War II, and particularly the second half of the 20^th^ century, there has been exponential growth in female participation in the labour market, essentially doubling between 1973 and 2002 [1]. In Canada, this long-standing trend has been the primary reason for increasing labour supply [7]. While the participation of women in labour markets peaked between the 1970s and 1980s, the trend continues to hold true to this day [8]. The increase participation in the labour market has been linked to several factors, including changes to family structure, economic pressures, and changes to social structures. Differences in women’s labour market participation across Canadian provinces have been attributed to variance in infertility rates and accessibility to day care, education and job opportunities. Accordingly, the management of dual roles of employee and caregiver - whether caring for dependent children and/or aging family/friends - has become of interest in the research and policy domains.

Women have traditionally been socialized to provide care for dependents, including elderly and younger family members; this gender role continues exist today. In fact, upwards of 85-90% of the care in Canada is provided by unpaid informal caregivers [9], and 80% of which are women [10]. Morris et al. [11] argues that “*women family members were expected to supplement home care services without pay and at greater personal expenses in terms of their own health, incomes, benefits, career development and pension accumulation, while men were not under as much pressure*…” (p. vi). Although more women are participating in the labour market, research indicates that their likelihood of becoming a caregiver has not diminished but, rather, has grown [12].

Although not exclusively a gendered issue, recent demographic and health service changes have created a new burden for female employees and caregivers. The number of terminally ill Canadians is expected to grow between two and four times from 2010 to 2030 [13], with the “baby boom” cohort nearing retirement and peaking between those years [14]. Many adults are being required to care for their aging parents in the community, often at home, while concurrently managing the responsibility of caring for their own children, a phenomenon that is expected to grow [15]. Known as the “third shift”, the caregiving shift is being negotiated alongside the first shift of paid labour, and a second shift of childrearing [16]. Social and moral attitudes have reinforced society’s expectations that family members will be end-of-life caregivers. The role of informal caregivers at end-of-life has also been heightened in response to financial pressure that has shifted health care from institutions into the community and private homes [13], together with desire to die at home [17]. These trends are reflective of the shifting neoliberalism we are experiencing in both culture and politics, all of which are proving to have a negative effect on the social construction of benefits, gender, and caregiving.

Despite the growing need for comprehensive policies, and the fact that quality end-of-life care has been identified as “the right of every Canadian”, end-of-life care has been described as a “patchwork quilt” [18]. As a result, the act of informal caregiving, and subsequent burden, has been described as an emerging public health issue. Although the rewards of caregiving are many, the costs of caregiving can be extensive; informal caregivers often report negative emotional, physical, social and psychological outcomes as a result of their caregiving duties [19-22]. Demands on caregiving also often translate into financial pressures; the average out-of-pocket cost of caregiving expenditures is often over $6,000 within the last month of life [23]. These out-of-pocket costs are particularly troublesome due to caregivers reporting decreased workplace productivity as a result of their caregiving roles [24].

Welfare state responses have evolved in order to meet these growing demands on employed workers, and particularly women, in recent years. These programs have been described as “*a set of government programs aimed at ensuring citizens’ welfare in the face of the contingencies of life…provid[ing] social insurance against the financial consequences of certain biological risks (illness, incapacity to work, childbirth, childrearing, old age)…”*[25]. The MLB and the CCB are two examples of welfare state responses aimed at supporting Canadian laborers’ and, in particular, woman’s multiple roles. The MLB was implemented in 1971 in recognition of the increasing number of women becoming involved in the paid labour force, and the necessity to implement policies to support women and develop equity in the workforce. The MLB applies to the case of children where the CCB applies to the case of all ages, but most commonly, elder adults. In 2004, a similar leave, entitled the Compassionate Care Benefit (CCB), was implemented in recognition of the informal caregiving work being required in light of the growing palliative and end-of-life population. Informal caregivers caring for families or friends at end-of-life can seek paid leave from work through the CCB. Both the MLB (including Parental Leave) and the CCB are legislated nationally through Employment Insurance (EI); applicants need to make contributions to the EI scheme in order to be successful in their application [26]. Contributions to the EI scheme occur through workplace payroll deductions, irrespective of the employees work status as either full-time or part-time, for example. While the financial supports available from both the MLB and the CCB are legislated through national policy, the individual provinces have jurisdiction beyond what is stipulated by the federal government; these are defined by provincial labour laws which, for example, guarantee women the right to take the MLB. National legislation is the sole focus herein. Differences between individual provincial and workplace policies, which may vary widely from the national standards, are not explored. The unique eligibility requirements and program features of both the MLB and CCB are summarized in Table 1.

**Table 1:** Eligibility requirements and coverage allotments of the maternity leave benefit and compassionate care benefit

**Maternity leave benefit**	**Compassionate care benefit**
*Eligibility requirements*	*Eligibility requirements*
- Demonstrate that your weekly earnings have been decreased by more than 40% (common eligibility requirement to all EI programs)	- Demonstrate that your weekly earnings have been decreased by more than 40% (common eligibility requirement to all EI programs)
- Have accumulated 600 insurable hours in the previous 52 weeks (qualifying period)	- Have accumulated 600 insurable hours in the previous 52 weeks (qualifying period)
- Provide the expected or actual date of birth of your child, if you are claiming the MLB;	- Have a doctor certificate documenting the care recipient is at risk of death within the next six months
- Provide your newborn's date of birth, or, when there is an adoption, your child's date of placement, if you are claiming Parental Leave Benefits. In the case of an adoption, you also need to provide the name and full address of the agency handling the adoption	- Be considered a family member, or “like” a family member
	- Have documented demographic information about the care recipient
*Benefit coverage*	*Benefit coverage*
- Must serve a two-week unpaid waiting period	- Must serve a two-week unpaid waiting period
- Upwards of 50 weeks of Benefits available, given a combination of Maternity and Parental Leave Benefits are taken	- Six weeks of paid Benefits (total length of leave is thus eight weeks)
	- You will receive 55% of your average insured earnings, to a maximum of $457/week
- You will receive 55% of your average insured earnings, to a maximum of $457/week	- Six weeks of leave can be split between multiple caregivers

Source: Service Canada (2010), through author’s compilation.

While the introduction of CCB marked a major step forward for supporting informal caregivers, low uptake rates suggests that the program has been unable to fulfill its mandate [27]. A 2008 EI tracking survey found that 49% of Canadians were not very aware, or not aware at all, of the CCB; this number has only dropped slightly from when the program was initially made available in 2004 [28]. The experience of the CCB contrasts with that of the MLB, which has shown higher uptake rates throughout the course of its evolution [29]. Canada’s MLB policy has received international acclaim as a result of its length and coverage, in particular its extension to fathers in the form of Parental Leave Benefits [30]. Of all those receiving EI in 2010, 29% took MLB or Parental Leave Benefits, while only 2% took CCB [26].

Reasons for the low uptake of CCB have been documented in earlier qualitative research [31]. In this work, the perspectives and expectations of front-line palliative care providers, caregivers and human resource personnel/employers was gathered; data analysis showed five key recommendations for improving the program and broadening its accessibility: (1) implementing a knowledge translation campaign to increase awareness; (2) simplifying and streamlining the application process; (3) eliminating the two-week unpaid waiting period; (4) increasing the length of the CCB, and; (5) increasing the financial remuneration available [31].

Due to their similarities in legislation and purpose, examining the evolution of the comparatively mature MLB may offer insights as to how the CCB may evolve in order to better meet the needs of informal caregivers. Identifying the processes through which changes to the MLB was implemented may suggest comparable mechanisms through which the CCB may develop. In order to do this, a review of the evolution of MLB and CCB policies was undertaken using Kingdon’s theoretical framework to evaluate policy change [6].

## Theoretical framework

Public policy involves the opening of 'policy windows', the development of policies, and their implementation and evolution. Kingdon offers a framework for understanding how issues gain a place on governmental agendas [6], which is relevant when trying to understand and explain the forces behind policy change. By using Kingdon’s framework, we simplify and analyze the context in which the MLB developed and evolved and consider the application of these results to the CCB [6]. In particular, Kingdon suggests that the opening of a policy window, which is a situation where agenda-setting and policy change can occur, happens through three channels: *problems*, *processes*, and *political processes*[6].

Kingdon defines *problems* as crises, prominent events, or changes in indicators that suggest the need to put an issue on the political agenda [6]. Metagora states that “*the rational behind this stream is that a given situation has to be identified and explicitly formulated as a problem for it to bear the slightest chance of being transformed into a policy*” [32]. *Processes* that can affect agenda-setting are defined as the gradual accumulation of knowledge or the development of policy proposals by specialists, including academic researchers [6]. The *processes* stream is concerned with “*the formulation of policy alternatives and proposals*” which may include competing policy proposals [32]. Finally, *political processes* denote changes in national mood and government, including; political priorities; political parties; party ideology; political context and/or; elections [6]. The political channel recognizes that “*political events, such as an impending election or change in government can lead a given topic and policy to be included or excluded from the agenda*” [32]. These three themes often connect in an iterative manner to create the opening of a policy window whereby changes can be made to legislation [6].

The application of Kingdon’s framework for policy agenda-setting has been explored in health service research before. For example, Exworth, Blane and Marmot used Kingdon’s framework to understand progress of health policy aimed at reducing health inequalities in the United Kingdom [33]. Sardell and Johnson described the implementation of a child health benefit policy in the United States through application of Kingdon’s framework [34]. Finally, Lantz, Weisman and Itani [35] have used Kingdon’s framework to understand the evolution of women’s health policy through the case of breast cancer screening. The use of Kingdon’s framework in this research has been chosen due to its accepted appropriateness for health policy research, together with its relative simplicity in tracing the evolution of policy ideas through channels which influence agenda-setting.

## Methodology

A review of changes to MLB policy was undertaken through analysis of media coverage. As tracing the origins of policy ideas involves an “infinite regress” [6], p. 72, ten years prior to the start of the MLB was chosen as a search starting point. Thus, the search for documentation of the evolution of the MLB covered the years between 1960 to present; this 50 year period was broken up into five year periods. The archival database of one of Canada’s national newspapers, *The Globe and Mail,* was searched given that it is the most comprehensive national newspaper archive available. The terms “maternity leave” and “maternity benefit” were searched, and the number of total hits for each period were recorded. A hit denoted the term being found in the article; thus multiple hits were recorded for each article. The archival system for the articles changed between 2005 and 2006 as the news articles were uploaded electronically in a different manner. Thus, for 2005–2010, the search of the terms did not yield both a number of hits and a total number of articles, but rather just pulled the article if the search term was mentioned at least once, without recording the number of times it was mentioned in each article. Each article was then reviewed through a directed content analysis [36], whereby the article was included for review and data extraction if it discussed the MLB in the context of its evolution. Articles were discarded if they were outside the scope of this analysis; for example, if they focused on events in other countries, provincial or workplace events without relation to national events, or were opinion pieces or letters to the editor, the latter which were deemed to be too subjective. Based on these inclusion/exclusion criteria, 163 of the original 2,698 articles were included. The articles saved were then analyzed closely, coded according to the three channels Kingdon identified (*problems*, *processes*, and *political processes*), and used to create a timeline of events and policy changes [6]. The codes were agreed upon by research team members to enhance rigour and validity (the number of hits for each of the three channels was counted to allow for comparison), following a summative content analysis approach [36]. Table 2 presents the quantitative results of the summative coding of the articles.

**Table 2:** Quantitative results of the summative coding of the articles

**Search term**	**Years**	**Number of hits (total times search term mentioned)**	**Number of pages (total number of articles with search term in it)**	Number of articles saved after review*	Number of articles coded as “process”*	Number of articles coded as “problem”*	Number of articles coded as “political process”*
Maternity Leave	January 1960 – December 1965	230	7	40	0	0	0
	January 1965 – December 1970	445	125	5	5	1	1
	January 1970 – December 1975	570	171	21	7	4	0
	January 1975- December 1980	474	156	5	3	4	1
	January 1980- December 1985	1,194	265	20	15	9	6
	January 1985 – December 1990	937	25	15	3	3	0
	January 1990- December 1995	666	210	10	3	9	1
	January 1995 – December 200	801	233	14	10	9	2
	January 2000- December 2005	874	242	11	6	7	1
	January 2005-December 2010	n/a	247	7	5	4	3
Maternity Benefit	January 1960 – December 1965	57	25	1	1	0	0
	January 1965 – December 1970	109	44	6	5	1	2
	January 1970 – December 1975	226	68	9	9	3	1
	January 1975- December 1980	166	57	5	2	4	1
	January 1980- December 1985	373	99	19	13	4	4
	January 1985 – December 1990	326	110	16	14	7	2
	January 1990- December 1995	207	76	2	0	2	0
	January 1995- December 2000	306	103	4	2	3	1
	January 2000- December 2005	286	88	0	0	0	0
	January 2005- December 2010	n/a	54	3	3	1	0
**Total**	n/a	2,698	163	106	75	n/a	26

*Certain articles may have been coded in multiple categories, resulting in total numbers for each category not adding up to reflect the total number of articles reviewed.

The evolutionary process of the MLB was then compared to the progress made by the CCB since its inception; this was accomplished by examining similar elements of the programs, together with the types of changes that have occurred since their legislation. Data on the evolution of the CCB was also collected through the media, although more varied than the media analysis undertaken for the MLB. A watching brief of media pieces and policy reports had been gathered since the program’s inception (2004), via frequent searches for new sources in media and publication search engines. The watching brief was used in the analysis of the CCB for its evolutionary changes, given the dearth of national media attention in newspapers. In addition, a directed content analysis [36] was also applied, using the same inclusion/exclusion criteria employed for the MLB search.

## Findings

The content analysis of MLB news media generated two layers of data: (1) an understanding of how the MLB evolved in Canada, as well as; (2) an understanding of what types of channels and pressures generated the opening of policy windows and the consequent evolution of the MLB.

### Evolutionary steps of the maternity leave benefit

The MLB available to Canadians have changed drastically since its inception in 1971. The earliest form of the MLB available spanned 15 weeks and was offered only to women. It operated as a measure to address inequalities in the workforce, where women were being laid off as a result of being pregnant. The most notable changes to the program have resulted in increased flexibility for applicants, with an extension of the program in the form of Parental Leave Benefits to biological fathers, adoptive parents, and recently, extension of the benefits to self-employed parents. The length of leave has been expanded greatly throughout the years, from an initial length of 15 weeks to its current amount of upwards of 50 weeks of leave, if a combination of maternity and Parental Leave Benefits is taken. The application process has also evolved to recognize women who are taking MLB for a second child soon after the first; the number of qualifying hours in that case has been reduced. Table 3 illustrates key events in the evolution of the MLB in Canada.

**Table 3:** Policy changes to maternity leave benefit

**Year**	**Event**
1971	15 week MLB introduced to biological mothers for those who have worked 20 weeks in the previous year. Through amendments to the Canada Labour Code, this also prohibited dismissal of lay-off because of pregnancy. Women needed to demonstrate that they had worked 10 weeks prior to their conception in order to be eligible (on top of regular Employment Insurance (EI)* eligibility criteria) - this was known as the “Magic 10 rule”.
1974	Flexible MLB was enacted, allowing women who qualified to collect 15 weeks of benefits at any point a 26-week period, for up to eight weeks prior to birth, and 17 weeks after.
1979	Employment Insurance amended to allow women who were on MLB in the previous year less than 20 weeks work in order to be considered eligible for MLB again.
1983-1984	Adoptive mothers awarded MLB, recognizing the purpose of the leave is not just for the physical recuperation of the mother, but also for the infant’s adjustment. Length of MLB granted is 15 weeks. 'Magic 10 rule’ was also abolished, requiring women to have only worked the mandatory amount for regular EI benefits.
1987	The federal government passed legislation making the MLB mandatory for employers under the Canada Labour Code, thereby closing a loop-hole.
1988	New legislation allowed mothers to delay the beginning of their MLB until they leave the hospital. Previous system had expired benefits after 15 weeks regardless.
1989-1990	Biological fathers were granted an additional 10 weeks leave on top of the 15 weeks of leave biological mothers are allotted through EI. The 10 weeks newly granted could be taken by the mother instead of the father as well. Sickness benefits were also be allowed to be taken on top of Parental Leave Benefits without penalty, and those who were out of work due to labour disputes could also have sought Parental Leave Benefits according to revisions to the Labour Code. Leave for adoptive parents was amalgamated into the same Benefit system, affording them the 10 weeks afforded to fathers, instead of the 15 weeks they were granted under employment laws in 1984.
1997	Change in the number of hours needed to qualify for the MLB from 300 to 700, meaning that 12,000 fewer women qualified in 1997, compared to 1996.
1999	Reforms to EI to form a 'special benefits’ program, which allowed parents to qualify for Parental (including Maternity) Leave Benefits with 600 insurable hours, rather than the previous demand of 700 insurable hours
2000	Many fathers were not taking |Parental Leave Benefits. In order to increase uptake policy changes increased the length of benefit to 35 weeks from 10 weeks, and also eliminated one of the two week waiting periods if both parents were applying (thus only one parent needed to the waiting period). EI also provided up to 55% of wages during 15 weeks of maternity leave- combined leave is therefore 50 weeks. Parents were also allowed to work part-time during Parental Leave Benefits, but not Maternity Leave Benefit.
2000	Liberal government announced plans to make it easier for a mother to apply for MLB if having a second child relatively soon after a first child by reducing the number of hours worked needed in order to be eligible. The number of hours worked needed for qualification was reduced to 600.
2009	Maternity Leave Benefit and Parental Leave Benefits were extended to self-employed workers, to be available in 2011. To be eligible, a self-employed worker must enter into an agreement with Service Canada one year prior to accessing the benefits.

*Employment Insurance was known as Unemployment Insurance in Canada until 1966, but is referred to as Employment Insurance throughout this table for consistency.

### Application of Kingdon’s framework to the maternity leave benefit

Analysis of the media coverage of the MLB from 1960, since pre-inception, and through to 2010 was accompanied by categorization of the specific events described in the media coverage according to the three policy channels identified by Kingdon [6]; *problems*, *processes*, and *political processes*. The number of articles accessed through the initial media search (*n* = 2,698), was much greater than those saved and categorized according to the three streams (*n* = 163) defined by Kingdon, as summarized in Table 2[6]. In addition, the three policy channels (*problems; processes* and; *political processes*) were quantified. It is important to note that often an article may discuss examples of multiple channels through which a policy change was influenced (i.e. it may discuss both a problem, as well as a process scenario, or may be considered both a prominent event as well as crisis). As such, while the quantification and tracking of the types of events can serve as a mechanism for general comparison, the actual numbers are more subjective. Even so, differences in the prevalence of each of the channels were evident. Once again, Table 2 presents the quantitative results of the summative coding of the articles. Certain articles may have been coded in multiple categories, resulting in total numbers for each category not adding up to reflect the total number of articles reviewed.

As Kingdon describes, *processes* include the gradual accumulation of knowledge and/or perspective among specialists in a given policy area, as well as the generation of policy proposals (106 process-type channels were categorized in the MLB media coverage) [6]. In particular, advocacy on behalf of academics, specialists, and stakeholders generated the most momentum in shaping MLB policy. For example, in 1967 the Women’s Bureau of Canada (Department of Labour) ran a MLB survey, showing that Canada was significantly behind international equivalents and discussed options for implementing a like policy. Similarly, in 1981, Federal Employment Minister Lloyd Axworthy tabled a report suggesting alternate policies that extended leave benefits to adoptive parents, a feature which was implemented in 1984. The generation of policy proposals such as these clearly represents the process route of agenda-setting. The opening of a policy window through a process route was not only undertaken by influential organizations or political figures, but also by academics. For example, in 1987, the national media covered academic research exploring the dual responsibilities faced by female employees, suggesting that an increase in the leave length of the MLB was necessary to support women managing multiple roles.

*Problems* were slightly less common as motivators for agenda-setting in MLB evolution (75 problem-type channels were categorized in the MLB media coverage). *Problems* are defined by Kingdon as 'crises', 'prominent events', and 'changes in indicators’ that generate incentive to change policy [6]. For example, the Schachter vs. Canada case influenced the implementation of Parental Leave Benefits for biological fathers in 1990. The Bliss vs. Canada case in 1994 also played a hand in mounting public pressure to abolish the 'Magic Ten’ rule. Previously women were required to prove they had worked ten weeks prior to conception in order to be eligible for the MLB, on top of regular eligibility criteria. 'Changes in indicators’ were also explored in the media coverage, and were also often categorized as processes, as they also usually represented gradual accumulation of knowledge by specialists and academics. For example, a study in 1993 reported that very few men were applying for maternity leave, despite the availability of Parental Leave Benefits. Changes were eventually made in 2000 that increased the length of leave available, with the hope that more leave time would motivate parents to split up caregiving duties. In addition, the two-week waiting period was amended, requiring only one parent to meet this stipulation, rather than two.

*Political processes* were by far the least common type of route through which policy windows were opened (26 political-process-type events were categorized in the MLB media coverage). Included in this category were 'swings in national mood’ and general 'changes to governance', such as elections, changes in administration and turnover in parliament. For example, in 1999, Jane Steward took over the role as the Human Resources Minister and was dedicated to improving benefits of the MLB due to her personal opinion that the first few years of a child’s life are critical, although many women returned to work directly after the benefit was over. Her influence prompted part of the interest in extending the length of leave. Similarly, political party platforms in the 2005 federal election brought about discussions and lobbying for EI changes, including Harpers’ promise to expand EI special benefits, such as the MLB and CCB, to self-employed.

Although there were significant differences in the type of route most often used to generate the opening of a policy window, additional trends were evident according to a temporal analysis of the evolution of the MLB. Processes were the most common type of route for agenda-setting prior to and during the earlier years of MLB legislation. For example, the first media discussions accessed about the MLB discussed Canada’s lack of benefits, and available research on international comparisons which could be used to generate a policy proposal. Problems became more common after the implementation of the MLB, and were influences by the involvement of female advocacy groups interested in minimizing inequality. Political processes, as by their definition, occur around governmental changes, an important contextual avenue which was not explicitly explored in this research but likely would demonstrate a temporal patterning in relation to national political changes.

The Compassionate Care Benefit (CCB), compared to the MLB, has had a relatively short evolutionary period. Since the initial legislation in 2004, the only policy change to the CCB occurred in 2006, when eligibility criteria changed to allow a more flexible definition of family member. Prior to this change, applicants were required to be a blood or law relation to the care recipient. New eligibility criteria allows the caregiver to be considered 'like family’ in response to the recognition that the care recipient should have flexibility in deciding who their more compatible caregiver is, and that the caregiver most suited to the role may be a neighbour or close friend, rather than a family member per se. In addition, the CCB has also recently been extended to self-employed workers, much like the MLB, to be available to applicants in 2011. Policy changes to the CCB have been summarized in Table 4.

**Table 4:** Policy changes to the compassionate care benefit

**Year**	**Event**
2004	CCB introduced was a six week paid leave from work in order to provide care for a dying loved one by a parent, child or spouse
2006	CCB eligibility criteria was amended to allow more family caregivers access to the program. New criteria requires the caregiver to be a providing care for a brother, sister, grandparent, grandchild, mother or father-in-law, uncle, aunt, niece, nephew, foster parent, ward, guardian or a person considered ’like family’
2009	Announcement made that the CCB will be extended to self-employed workers in 2011. To be eligible, applicants must reach an agreement through Service Canada one year prior to accessing the CCB.

## Discussion

The results indicate a number of ways in which the MLB has evolved to better meet the needs of female caregivers in Canada. As well, Kingdon’s framework identified possible mechanisms through which those policy changes were able to happen [6]. As such, two findings can be made in comparing the evolution of the MLB with that of the CCB: first, research results potentially indicate the types of changes that may be forthcoming for the CCB; second, results indicate how best to effectively generate the opportunity for changes to happen via the opening of policy windows. The developmental changes made to the MLB have allowed it to be more accessible and comprehensive and informs the potential development of the CCB. Four recommendations for the CCB have been derived from the MLB findings. If implemented, they may allow the CCB to better support informal caregivers at end-of-life. These have been summarized in Table 5 and are discussed in detail below.

**Table 5:** Key policy recommendations for CCB based on a comparison to similar MLB features

**Key feature of MLB endorsed**	**Comparable feature currently available through CCB**	**Policy recommendation(s) for CCB**
Recommendation A		
● Introduction of longer MLB lengths (upwards of 50 weeks)	● Eight weeks of CCB leave allocated, with only six weeks of financial compensation	● Increase the length of CCB leave, possibly to the length recognized as the end-of-life period by Service Canada (the last six months of life)^1^
● Increases in MLB lengths at several points during the evolution of the policy		
Recommendation B		
● Flexibility in when the MLB leave is taken	● CCB leave must be taken in the six months prior to death, and end with death of the care recipient, regardless of how much of the eight weeks of leave was taken	● Allow caregivers to carry some of the CCB leave over to the bereavement period to allow them time to grieve and manage funeral arrangements
● MLB may be split up before and after birth or adoption		
Recommendation C		
● Flexibility in option for multiple applicants	● Only eight weeks of CCB leave are assigned for each care recipient, regardless of the number of caregivers splitting the leave	● Allow multiple applicants each their own CCB leave, instead of limiting the leave to one per care-recipient
● Additional leave given for biological fathers and adoptive parents		
Recommendation D		
● More generous application process for mother’s applying a second time within a short period	● No special consideration for caregivers called upon to provide end-of-life care and support multiple times within a short time period	● Reduce the number of qualifying hours needed for applicants who are applying again within a short time after the first leave was taken
● Reduced number of hours needed to qualify		

^1^Service Canada (2009).

### Recommendation A

At several points during its evolution, the MLB has expanded its coverage length to allow longer lengths of leave in response to social changes. Adoptive mothers were granted 15 weeks of leave originally, in understanding that the purpose of maternity leave is not just for the physical recuperation of the mother, but also for the newborn or child’s adjustment. In 1989 an additional 10 weeks of leave on top of the 15 weeks of leave were granted to allow biological fathers time to bond with the child and partake in caregiving. The additional 10 weeks of leave were made available to either natural mothers or fathers. Later, changes in 2000 extended the length of leave available again, in part due to the recognition that many men were not taking Parental Leave Benefits. The new regulations increased the length of Parental Leave Benefits to 35 weeks (from 10 weeks), making the total length of the MLB and Parental Leave Benefits together 50 weeks. Additionally, the waiting period was eliminated for one parent if both parents were applying.

### Recommendation B

In 1974 the MLB was made more flexible by allowing women to collect 15 weeks maternity leave at any point during a 26 week period, including eight weeks prior to, and up to 17 weeks after, birth. In contrast, the CCB only allows for six weeks of leave, and only prior to death. Regardless of how many weeks of leave the applicant has actually used, the leave ends following the death of the person being cared for; this is extremely limiting, given the period of bereavement and grieving that may follow end-of-life. As Hallenbeck states, “*care does not end with death of the patient but continues through death pronouncement, family notification of the death, discussion of autopsy, and immediate bereavement support.*” [37], p. 2265. Bereavement and grief has been reported to take between six months [38] to 24 months [39], although quantification is difficult [39]. Negative components of grief, including anger and depression, often do not peak until five or six months after death [39]. Indeed, attention to the care needs of the informal caregiver after the patient’s death should be built into the CCB, given that the caregiver is part of the unit of care [40,41].

### Recommendation C

The Compassionate Care Benefit (CCB) has also recognized the need to allow for multiple caregivers, both by allowing multiple people to apply for and split the eight week leave and by changing its definition of eligible family members in 2006 to include those considered 'like family’. However, unlike the MLB, the CCB has not been extended; only eight weeks are available to be split between caregivers. Considering difficulties in prognostication at end-of-life, extending the length of the CCB needs to be considered. Prognostication at end-of-life is complex, as it includes not only estimating time until death, but also informs the disease trajectory, all of which impacts: care receiver symptoms; caregivers’ life worlds; financial costs, and; changing therapies [42]. Both the prediction of the disease trajectory, as well as prediction of time-of-death, are difficult, and have been reported as potential barriers to timely access to hospice palliative care [42,43]. Underutilization of hospice palliative care services has been associated with poor prognostication and palliative care referral [44,45]; this underutilization may impact those eligible for the CCB, given the limited time frame in which they are considered eligible, and given their self-reported difficulty in deciding when to apply in order to maximize their time spent with the patient [31]. By lengthening the amount of CCB leave available, these issues specific to prognostication and concerns about when to take the leave may be negated.

### Recommendation D

The Maternity Leave Benefit (MLB) has incorporated, during the course of evolution, another key feature which introduces more flexibility in its eligibility criteria; this would also be appropriate for the CCB. In 1979, and again in 2000, eligibility criteria were eased for women who had been on Maternity Leave in the previous year and who were applying for Maternity Leave again, allowing them to have worked fewer hours in order to qualify. While some research indicates that women do not increase their participation in the labour market in the year preceding birth, in order to be eligible for the MLB [1], other research indicates that availability of the Canadian MLB policy may influence a couples’ decision to pursue parenthood [1,46,47]. Similarly, fertility planning around maternity leave policies has shown strong correlation in international research [48,49].

Informal caregivers at end-of-life may also be required to provide palliative care repeatedly in one year. We know, for example, that hospitalization or death may cause negative health outcomes in another who is closely connected [50]. Bereavement has been associated with increased risk of mortality, particularly in the first six months after a loved-one’s death and most common among spousal deaths [51]. Therefore, it is likely that caregivers may be called upon to provide palliative care more than once a year. Creating more flexible eligibility criteria for end-of-life caregivers, similar to the MLB, would provide more comprehensive coverage to Canadians.

Triangulating the results of tracking the evolution of the MLB, together with the temporal patterning of how policy windows are opened, generates some important information that can inform potential changes to the CCB. Considering that *processes* were the most common measures identified in this study, through which policy windows were opened and positive changes to MLB legislation was achieved, it follows that perhaps resources and energy should be directed via stakeholders, specialists and academics at *processes* to facilitate the opening up of policy windows.

Identification of key stakeholders in end-of-life care, and focused campaigning on their behalf may prove to be the most effective way to generate policy changes to the CCB. Indeed, this has already been witnessed. For example, the CCB, as a policy proposal, was first generated due to the advocacy of community stakeholders, including invested government officials like Senator Sharon Carstairs who, in 2000, published her report *Quality End of Life Care: The Right of Every Canada.* Other stakeholder groups are already advocating for more flexible coverage, including increasing the length of the leave. For example, the Canadian Cancer Society put forth a news release in 2008 discussing the results of a poll, which showed the majority of caregivers wanted a more comprehensive program that entitled them to at least six months of caregiver leave from work.

*Problems*, according to the results of MLB analysis, could perhaps be capitalized upon later in the evolution of the social program, once they have had the opportunity to arise. Interestingly, a case was presented to the Board of Referees in 2005 in which a women was denied access to the CCB to care for her dying sister as she was not recognized as a 'family member’ under the original eligibility criteria. The case prompted the statement by the Board who, although forced into upholding her denied claim, said:

*The Board finds no that there is no compassion…the failure of the Commission and the Minister to act swiftly in these matters of Compassionate Care amendments has only served to exacerbate the suffering endured by families as they care for a dying family member…*[52].

Having gathered national media attention and, together with the *process*-type stakeholder advocacy, this *problem*-type event prompted the later opening of a policy window to change the eligibility criteria to be more inclusive. Eligibility criteria now allow applicants from a variety of relationships to the care recipient to apply, including those considered 'like family’.

However, the CCB is nine years past implementation, yet very few problems have reached the mainstream media or generated momentum for policy change. One hypothesis for this is the general disinclination to discuss end-of-life care; Canada remains largely death-denying, with a greater focus on curative medicine than on comprehensive care [53]. Greater efforts to bring problems with hospice palliative care services, including the CCB, into the mainstream will force national attention on these issues, and perhaps generate more political will among government to add hospice palliative care and the CCB to their political agendas. These efforts may gain momentum given that a growing number of Canadians face the realities of providing end-of-life care, reflected in the aging of the 'baby boomer’ generation. More focus on the *process* and *problem* realms of agenda setting may, in turn, generate more impetus for taking up the cause of palliative care by politicians, thus setting up opportunities for agenda-setting. Further, there exist salient differences between the MLB and CCB. For example, the MLB has two long-term economic goals, those being: (1) the retention of women in the labour force, and; (2) producing healthier and well-adjusted babies; without the MLB, Canadians may not have babies. In contrast, the CCB shares only one of these goals: that being retaining women in the labour force.

While evidence about the influence of stakeholders and champions in moving policy forward has been explored in the literature, it is also noted that the process of opening policy windows is a complex and iterative process. As Kingdon notes, it is the coupling of one or more of the three streams that opens policy windows [6]. Interestingly, the slow temporal nature of policy change for both the MLB and CCB is perhaps indicative of the Canada’s policy system. Canada has been labeled as having an incremental policy system, where policy changes occur without an overarching framework, and where responsibility for changes in the health care system falls to both the provincial and federal level governments [54].

By implementing some of the policy recommendations identified, the CCB will better support the needs and expectations of informal caregivers and allow women, in particular, a better chance for success in balancing multiple roles and thereby enjoy better health and wellbeing [55]. There is no question that this issue will likely become a greater concern to women in the coming years, as the number of people requiring care at end-of-life continues to grow [18].

### Limitations

A number of limitations have been identified in this research, particularly with the application of Kingdon’s framework, as well as the methodological approach. As Kingdon discusses, tracing the origin of policy ideas is an iterative, never-ending process [6]. Thus, the setting of an arbitrary time point at which to start the literature search, while necessary, may have limited earlier events important to the evolution of the policies investigated. The use of *The Globe and Mail* newspaper as the sole source for media coverage of the MLB may limit the scope of the media coverage reviewed. For example, while *The Globe and Mail* typically represented national events, articles with provincial foci were often about Quebec and Ontario, leading to concern that events in other provinces may not be adequately reviewed. As the focus of this paper is national, this is of minimal concern, however there is the potential for events in other provinces, which may have influenced agenda-setting, to have been missed. Related to this is the exclusion of associated provincial policies. Further, the exclusion of a wide range of other sources, such as, for example: primary documents made by proponents of legislative change; other media sources, or; actual statements by politicians, other policy makers, or members of civil society not filtered through the media. In addition, as the search terms used for the whole time period were “maternity leave” and “maternity benefit” the search strategy did not take into account articles about employment insurance generally that may have affected the evolution of maternity leave. For example, changes to employment insurance according to economic pressures and political changes may have influenced changes in eligibility and coverage available to MLB applicants. Similarly, the search terms did not include “Parental Leave Benefits” or “paternity leave”, which may have reduced the number of relevant hits if the article did not use the term “maternity” as well. Finally, there are some limitations in choosing Kingdon’s framework; policy windows are only one part of the discourse on policy development and other frameworks and theories were not considered, although it is recognized that they could provide different perspectives [56]. Likewise, Kingdon’s framework does not account for the magnitude an event will have or distinguish significance between two or more different events [6]. For example, we cannot know the impact of one academic report versus another. Further, it is likely that, given the retrospective analysis, contextual factors such as the political landscape and the socio-economic climate of the time in which policy windows were opened, have not been fully accounted for in this analysis.

### Future research directions

Both the MLB and CCB have, in fact, been the focus of attention recently, as governments examine the opportunity to separate both policies from the Employment Insurance program, and whether to devolve the responsibility to provincial levels [1]. Much of the interest of in doing so has developed in light of the separation of such policies from the federal government in the province of Quebec. Quebec has run its own family policy system separate from the national system since 2006, and has reported huge success in increasing support systems through the evolution of the provincial policies [1]. Indeed, while not examined, much of the media attention initially picked up through MLB and CCB reviews covered the separation of Quebec benefits. Similarly, workplace policies, and lobbying for enhanced workplace benefits was a continuous topic of interest in the media. For example, media coverage of the MLB in the 1980s focused largely on the Canadian postal workers’ fight for the MLB. It is likely their experiences may have shaped ensuing national policy. Closer examination of these events, and their potential for opening up policy windows, would be valuable. Further, linking the developmental changes experienced by both the MLB and CCB with actual changes in uptake would be most valuable. If this were accomplished, responses to programmatic changes could be known and used to inform future program revisions.

## Conclusion

The evolution of Maternity and Parental Leave Benefits in Canada has also been studied by Phipps [1] via an examination of the social and economic contexts throughout the history of the MLB. While Phipps’ research has been valuable in setting the context for this study and aiding in fact-checking, the research presented herein presents a novel examination of MLB evolution according to Kingdon’s framework, providing an analytical comparison with the CCB [1,6].

The question of how an issue gains importance on a political agenda is an important one. Women’s health researchers, advocates and policy-makers may consider applying a similar framework for understanding this phenomenon. As the population continues to age worldwide and more women are called upon to become informal caregivers, those in other countries have begun to look to Canada as a leader in developing informal caregiving policies. The findings of this paper have international scope for those interested in developing policies similar to the CCB, both in identifying venues through which they may be introduced into governmental discussions, as well as showcasing possible policy concerns and features. The application of Kingdon’s framework has been discussed clearly within this paper. Given the frameworks simplicity, and relevance to describing how policies are generated, others may consider a similar approach to researching women’s health policy-making.

Women’s health and wellbeing has been intricately linked to social policy given women’s economic vulnerability, together with their societal roles as caregivers to children, relatives and friends [57]. Social assistance policies, employment insurance programs, and health and social services, as components of the welfare state, are implicated as determinants of women’s health in Canada [57]. Exploring how to improve such benefits, as attempted herein with respect to the CCB, is extremely important given their impact on health, and women’s health in particular.

In comparing the CCB to MLB, it is evident that the CCB has already mimicked some of the evolutionary processes, having expanded its eligibility criteria to allow more applicants to seek benefits. However, the CCB must undergo further refinement before being truly supportive for informal caregivers at end-of-life, such as lengthening the amount of leave available. Given that the results of MLB analysis show that *processes -* namely the advocacy work by specialists, stakeholders, and academics, may be the most effective way of opening policy-windows and having events placed on the political agenda in the evolution of the MLB, it is hypothesized that the process route may prove equally valuable in agenda-setting for the CCB.

## Abbreviations

CCB: Compassionate care benefit; MLB: Maternity leave benefit.

## Competing interests

The authors declare that they have no competing interests.

## Authors’ contributions

SD undertook the data collection and analysis. AW oversaw the overall study, cross-checked the data analysis, conceptualized the paper design, while providing numerous editorial comments. Both authors read and approved the final manuscript.

## Authors’ information

SD recently received her Master’s degree from McMaster University and is now studying nursing at Trent University. AW is an Associate Professor at McMaster University leading a research program in various sub-fields of social and health geography.

## Pre-publication history

The pre-publication history for this paper can be accessed here:

http://www.biomedcentral.com/1472-6874/14/60/prepub
